# Is Preoperative Clopidogrel Resistance a Predictor of Bleeding and Risks in Patients Undergoing Emergency CABG Surgery?

**DOI:** 10.21470/1678-9741-2018-0005

**Published:** 2018

**Authors:** Mehmet Kizilay, Zeynep Aslan, Unsal Vural, Ahmet Yavuz Balci, Ahmet Arif Aglar, Sahin Yilmaz

**Affiliations:** 1 Department of Cardiovascular Surgery, Dr. Siyami Ersek Training and Research Hospital, University of Health Sciences, İstanbul, Turkey.; 2 Department of Cardiovascular Surgery, Derince Training and Research Hospital, University of Health Sciences, Derince, Kocaeli, Turkey.; 3 3 Department of Anesthesiology, Dr. Siyami Ersek Training and Research Hospital, University of Health Sciences, İstanbul, Turkey.

**Keywords:** Platelet Aggregation Inhibitors, Thienopyridines, Coronary Artery Bypass, Acute Coronary Syndrome

## Abstract

**Objective:**

The aims of this study were to determine whether the detection of preoperative clopidogrel resistance in patients undergoing cardiac surgery while using clopidogrel could play a guiding role in the prediction of postoperative excessive bleeding, transfusion requirements, and risks and to provide clinically significant data.

**Methods:**

Two hundred and twenty-two patients [median age: 59.4 (38-83) years; 38 females] undergoing emergency and elective coronary artery bypass graft (CABG) surgeries in our clinic were evaluated prospectively. Patients with multiple systemic diseases, other than diabetes mellitus (DM) and hypertension (HT), were excluded. Patients receiving clopidogrel were also evaluated for clopidogrel resistance and grouped according to the results of this test. Assessments of platelet functions were performed by multiplate impedance aggregometry method and adenosine diphosphate test.

**Results:**

The use of postoperative fresh blood replacement and platelet transfusion was higher in patients receiving clopidogrel than in those not receiving it (*P*=0.001, *P*=0.018). DM, HT, myocardial infarction, and the number of presentation to the emergency room were significantly higher in patients receiving clopidogrel than in those not receiving it (*P*<0.05). No significant difference was determined between patients with and without clopidogrel resistance regarding the amount of bleeding during and after surgery, erythrocyte suspension and fresh-frozen plasma transfusion rates, preoperative troponin values, ejection fraction values, and length of hospital stays (*P*>0.05).

**Conclusion:**

We think that resistance studies in patients receiving clopidogrel before cardiac surgery are not efficient to predict bleeding and bleeding-related complications in patients undergoing emergency and elective CABG surgeries.

**Table:** 

Abbreviations, acronyms & symbols				
ACS	= Acute coronary syndrome		ERT	= Electrophoresis release test
ADP	= Adenosine diphosphate		FFP	= Fresh-frozen plasma
ASA	= Acetylsalicylic acid		Hb	= Hemoglobin
AUC	= Area under the aggregation curve		HT	= Hypertension
BARC	= Bleeding Academic Research Consortium		ICU	= Intensive care unit
CABG	= Coronary artery bypass graft		INR	= International Normalized Ratio
CPB	= Cardiopulmonary bypass		IQR	= Interquartile range
CURE	= Clopidogrel in Unstable angina to prevent Recurrent		LIMA	= Left internal mammary artery
	ischemic Events		MI	= Myocardial infarction
DAPT	= Dual anti-platelet therapy		PCI	= Percutaneous coronary intervention
DM	= Diabetes mellitus		RIMA	= Right internal mammary artery
EF	= Ejection fraction		STEMI	= ST-elevation myocardial infarction

## INTRODUCTION

Clopidogrel is a thienopyridine derivative adenosine diphosphate (ADP) receptor blocker which inhibits platelet aggregation specifically and irreversibly^[1]^. The effect of clopidogrel begins within 2 hours after oral administration and lasts for 5-7 days. The time required to both the platelet inhibition response and the antiplatelet activity return to normal levels varies. Due to its antiplatelet effect, clopidogrel is used to prevent ischemic events in patients with acute coronary syndrome (ACS) and before percutaneous coronary intervention (PCI)^[2]^. Approximately 10-15% of ACS patients undergo coronary artery bypass graft (CABG) surgery since they are not suitable for PCI. In addition, patients with unfavorable coronary anatomy for PCI and those who underwent failed PCI become candidates for emergency CABG surgery^[3]^. In most of these patients, one thing is certain: the antiplatelet agent, particularly clopidogrel, used before PCI and hence is considered to be responsible for postoperative bleedings and it has been a controversial subject for a long time^[4]^. In current guidelines, it is recommended to cease clopidogrel 5-7 days before surgery in patients who will undergo elective CABG surgery^[5,6]^. The clinical benefits of using dual anti-platelet therapy (DAPT) with acetylsalicylic acid (ASA) and P2Y12 inhibitors in patients with ACS or after PCI have been well established. Premature discontinuation of antiplatelets before CABG surgery increases the risk of a thromboembolic event, while the continuation of antiplatelets increases the risk of bleeding. However, in such conditions, it is not clarified exactly which antiplatelet agent, as a drug companion to ASA, should be used or when it should be discontinued^[7]^.

Although it is reported in some articles that emergency conditions do not cause additional CABG complications, there are some articles that point to an increase in postoperative complications of CABG surgery such as bleeding, cardiac tamponade, reoperation, and excessive blood transfusion under emergency conditions^[8]^. Unfortunately, there is no algorithm to evaluate the benefits and risks on this subject and which aims to solve this problem.

Another issue related to clopidogrel is the resistance. The rate of clopidogrel resistance vary between 4% and 30% in current literature^[9]^. Because of this resistance, efficient antiplatelet treatment cannot be provided to some patients despite the use of clopidogrel and this may cause complications such as stent and graft thrombosis and myocardial infarction (MI). On the other hand, the possibility of a considerable amount of resistant cases suggests the question: "Is the predetermination of resistance to clopidogrel in patients who will undergo CABG surgery while receiving clopidogrel a guide for conditions such as possible postoperative bleeding and related complications?". Considering this condition, in our study, we investigated complications of clopidogrel such as the amount of bleeding during and after CABG surgery, use of blood and blood products, tamponade, and reoperation. Also, because of the prevalence of clopidogrel resistance, we decided to perform this study to investigate the effect of the identification of patients with and without clopidogrel resistance on the use of blood and blood products and postoperative complications.

## METHODS

Two hundred and twenty-two patients [median age: 59.4 (38-83) years; 38 females] undergoing emergency and elective CABG surgeries in our clinic, between January 2015 and July 2016, were evaluated prospectively. Patients were included in the study after receiving medical and ethical approvals. They were divided into two groups: patients undergoing emergent or urgent surgery while receiving clopidogrel preoperatively [n=111; median age: 58 years (38-83); 13 females] and patients undergoing elective surgery without clopidogrel use preoperatively [n=111; median age: 61.8 years (42-85); 28 females]. The patients receiving clopidogrel were also grouped inter se as those with clopidogrel resistance and those without it. The onset date of follow-up of patients for ACS in the emergency room was the inclusion date in the study; hospital discharge date after surgery was the exclusion date from the study. Patients with heart valve surgery, ventricle restoration, aortic surgeries, patients undergoing reoperation with bleeding disorder, thrombocytopenic patients, patients with renal failure, dialysis-dependent patients and patients with multiple systemic diseases [except hypertension (HT) and diabetes mellitus [(DM)] were excluded from the study.

In this study, patients were compared in terms of age, gender, DM, HT, ejection fraction (EF), time of surgery (emergent or elective), hemoglobin (Hb), platelet, International Normalized Ratio (INR), and troponin values in the preoperative period. They were also compared regarding cardiopulmonary bypass (CPB) and cross-clamping times in intraoperative period and bleeding, tamponade and reoperation rates, use of homologous blood and blood products, drain removal, length of intensive care unit (ICU) stay, and hospitalization in postoperative period. Transfusion decision was made after a thorough examination of the patient's clinical status and based on amount of bleeding, Hb (cut-off value: 8-9 mg/dL) level, and hematocrit (cut-off value: 24-27%) level.

### Surgical Intervention

Median sternotomy and standard CPB techniques were used in all the patients. CPB was established with aortocaval cannulation; myocardial protection was ensured with 30-32˚C systemic hypothermia and intermittent antegrade cold blood cardioplegia administered at 20-minute intervals. After cross-clamping and primarily distal anastomoses were performed; following removal of cross-clamping, secondarily proximal anastomoses were performed under partial cross-clamping.

The patients' postoperative bleedings were evaluated. Bleedings occurring within the first postoperative 48 hours were evaluated as postoperative bleedings. Bleeding Academic Research Consortium (BARC) criteria were considered for CABG-related bleeding^[10]^. Development of cardiac tamponade and volume of chest tube drainage >600 mL within the first hour or ≥200 mL/h for 3 hours were criteria for reoperation. Patients' drains with ≤100 mL serous drainage for 4 hours were removed during subsequent drain follow-ups. The patients were followed-up in the ward after an average follow-up of 1 day in ICU. Patients in good condition were discharged on postoperative day 5-7.

Multiplate(r) (2013 Roche Diagnostics International Ltd. CH-6343 Rotkreus Switzerland) impedance aggregometry method was used for platelet reactivity studies in patients receiving clopidogrel*.* The test was performed by taking a fasting blood sample on the first postoperative morning. Blood samples were taken into blood collection tubes containing hirudin as the anticoagulant agent. ADP test (2012 Roche Diagnostics GmbH Sandhofer Strasse 116D68305 Mannheim) was performed. Aggregation procedure was performed within 2 hours after taking a sample. The area under the aggregation curve (AUC/minute) was calculated with multiplate aggregometry measurements. Values of 0-200 AUC/min, 200-425 AUC/min, and 425-998 AUC/min were considered to indicate 80% sensitivity to the drug, dose that should be arranged, and resistance to the drug, respectively.

### Statistical Analyses

Continuous variables were expressed as mean (±) standard deviation and categorical variables were expressed as number and percentage (%). For intergroup comparisons of continuous variables, Shapiro-Wilk test was used regarding the conformity of the data to a normal distribution. Mann-Whitney U test was used for comparisons of data without normal distribution between groups. Categorical or nominal variables were analyzed by using Yate's corrected chi-square test and Fisher's exact chi-square test. The effect of preoperative demographic characteristics on postoperative complications was investigated by using Yate's corrected chi-square test and Fisher's exact chi-square test. Spearman's correlation test was used for comparison of the correlations between groups. Data observed to have a relationship were investigated by logistic regression analysis. Values of *P*≤0.05 were significant. SPSS statistical program (SPSS for Windows, version 20.0, SPSS Inc, New York, USA) was used for statistical analysis.

## RESULTS

The patients were divided into two groups as patients receiving and not receiving clopidogrel preoperatively. Additionally, the patients receiving clopidogrel were also divided into two groups as those with clopidogrel resistance and those without it. Distribution of the cases according to demographic features was shown in Table 1. DM, HT, MI, and the number of presentation to the emergency room were significantly higher in patients receiving clopidogrel than in those not receiving it (Table 1; *P*<0.05). Similarly, a significant difference was determined between troponin (*P*=0.029) and Hb levels (*P*=0.03). However, no significant difference was determined between preoperative EF values (*P*=0.088) and platelet and INR levels (*P*=0.175, *P*=0.128).

**Table 1:** Statistical analysis of preoperative demographic characteristics of the cases.

		Use of clopidogrel				*P*
		Absent		Present		
		n	%	n	%	
Gender	Female	25	66	13	34	0.032^b^
	Male	86	47	98	53	
DM	Absent	59	44	76	56	0.019^b^
	Present	52	60	35	40	
HT	Absent	39	37	66	63	<0.001^b^
	Present	72	62	45	38	
MI	Absent	89	61	58	39	<0.001^b^
	Present	22	29	53	71	
Angiography	Emergency	12	28	31	72	0.001^b^
	Elective	99	55	80	45	
Surgery	Emergency	7	22	25	78	0.001^b^
	Elective	104	55	86	45	
		**Median**	**IQR**	**Median**	**IQR**	
Age (year), mean±sd		60.41	9.42	58.34	9.28	0.100^c^
Preoperative EF (%)		55	45, 60	50	45, 60	0.088^a^
Troponin (ug/L) (n= 135)		0.05	0.01, 0.85	0.36	0.04, 1.25	0.029^a^
Preoperative Hb (g/L)		13.3	12, 14.2	13.8	12.5, 14.9	0.030^a^
Preoperative Plt (thousand/µL)		238	200, 289	217	184, 283	0.175^a^
Preoperative INR		1.02	0.96, 1.1	1.04	1, 1.08	0.128^a^

a Mann-Whitney U test;

b Pearson's chi-square test;

c independent samples t-test.

DM=diabetes mellitus; EF=ejection fraction; Hb=hemoglobin; HT=hypertension; INR=International Normalized Ratio; IQR=interquartile range, reported as first quartile, third quartile; Mean ± sd =Mean ± standard deviation; MI=myocardial infarction; Plt=platelet *P* values. A *P* value of <0.05 was considered significant.

When postoperative findings were evaluated (Table 2), no difference was determined between groups regarding the use of protamine sulfate, but the use of tranexamic acid (10 mg/kg) was higher in patients receiving clopidogrel than in those not receiving it, although it was not significant. No statistically significant difference was determined between the numbers of reoperations (*P*=0.553). The volumes of postoperative fresh blood and platelet replacement were significantly higher in patients receiving clopidogrel than in those not receiving it (*P*=0.001, *P*=0.017). Between the groups receiving and not receiving clopidogrel, no statistically significant difference was observed in postoperative EF levels, drain removal periods, types of graft used in CABG surgery [left internal mammary artery (LIMA) or right internal mammary artery (RIMA)], length of ICU stay, and hospitalization periods (*P*>0.05; Table 2). Use of clopidogrel showed correlation at a rate of 26% with whole blood replacement (*P*=0.01, r^2^=0.26). Use of tranexamic acid showed correlation at a rate of 12% with use of clopidogrel (*P*=0.076, r^2^=0.12). Namely, while the use of clopidogrel was moderately correlated with blood replacement, it was weakly correlated with use of tranexamic acid.

**Table 2:** Analysis of postoperative clinical data according to use of clopidogrel.

		Clopidogrel				*P*
		Absent		Present		
		n	%	n	%	
Protamine sulfate	Absent	96	49	100	51	0.404^b^
	Present	15	58	11	42	
Tranexamic acid	Absent	92	53	81	47	0.075^b^
	Present	19	39	30	61	
Reoperation	Absent	104	50	106	50	0.553^b^
	Present	7	58	5	42	
Pericardial effusion (n= 71)	Absent	5	20	20	80	0.566^b^
	Present	12	26	34	74	
Tamponade (n= 71)	Absent	15	23	51	77	0.587^d^
	Present	2	40	3	60	
LIMA/RIMA (n= 211)	LIMA	102	49.5	104	50.5	0.683^d^
	RIMA	3	60	2	40	
Saphenous graft	Used	103	92,8	110	99.1	0.035^a,d^
	Not used	8	7,1	1	0.9	
Use of platelet transfusion (unit)	Present	2	1,8	10	9.0	0.018^a^
	Absent	109	98,2	101	91.0	
Fresh blood replacement (unit)	Present	16	14,4	41	36.9	0.001^a^
	Absent	95	85,6	70	63.1	
		**Median**	**IQR**	**Median**	**IQR**	
Bleeding (mL)		300	200, 500	300	200, 500	0.987^a^
CPB time (minute)		97	77, 116	89	74, 110	0.248^a^
Cross-clamping time		59	45. 78	53	42, 66	0.106^a^
Number of bypasses		3	2, 3	3	2, 4	0.207^a^
Postoperative bleeding (24h)		750	600, 1000	750	500, 1100	0.680^a^
Amount of postoperative drainage		1000	700, 1350	1000	700, 1450	0.778^a^
Erythrocyte (unit)		0	0, 1	0	0, 1	0.533^a^
FFP (unit)		2	0, 2	2	0, 2	0.100^a^
Postoperative platelet number		170	147, 208	179	143, 227	0.216^a^
Postoperative EF (%) (n= 71)		50	45, 55	50	45, 60	0.160^a^
Drain removal (day)		2	2, 3	2	2, 3	0.621^a^
Intensive care (day)		1	1, 2	1	1, 1	0.706^a^
Clinical stay (day)		6	5, 6	6	5, 6	0.429^a^
Hospitalization (day)		7	6, 7	7	6, 8	0.477^a^

a Mann-Whitney U test;

b Pearson's chi-square test;

d Fisher's exact test.

CPB=cardiopulmonary bypass; EF=ejection fraction; FFP=fresh-frozen plasma; IQR=interquartile range, reported as first quartile, third quartile; LIMA=left internal mammary artery; RIMA=right internal mammary artery

During comparison of patients with and without clopidogrel resistance, among the ones receiving clopidogrel, no significant difference was determined between troponin values, preoperative and postoperative EF values, total bleeding volumes during and after surgery, amount of erythrocyte suspension [electrophoresis release test (ERT)] and fresh-frozen plasma (FFP) transfusions, and length of hospitalization (Table 3; *P*>0.05). However, lengths of ICU stay and cross-clamping times of patients without clopidogrel resistance were statistically significant (Table 3; *P*=0.020; *P*=0,040). No correlation was observed between clopidogrel resistance and CPB times (Table 3; *P*>0.05). Presence of clopidogrel resistance did not affect the numbers of effusions and tamponades developing during the follow-up period (Table 3; *P*>0.05).

**Table 3:** The effect of presence or absence of clopidogrel resistance in the patients on postoperative events and clinical medications.

		Clopidogrel resistance				*P*
		Absent		Present		
		n	%	n	%	
Effusion	Absent	5	20	20	80	0.566^b^
	Present	12	26.1	34	73.9	
Tamponade	Absent	15	22.7	51	77.3	0.587^d^
	Present	2	40	3	60	
LIMA/RIMA	LIMA	15	22.1	53	77.9	^‡^-
	RIMA	0	0	0	0	
Saphenous graft	Used	45	100	26	100	
	Not used	-	-	-	-	
Use of platelet transfusion (unit)	Present	0	0	3	5.6	0.321
	Absent	17	100	51	94.4	
Fresh blood replacement (unit)	Present	1	5.9	23	42.6	0.005^a^
	Absent	16	94.1	31	57.4	
		**Median**	**IQR**	**Median**	**IQR**	
Troponin (ug/L)		0.36	0.01, 0.55	0.33	0.06, 1.7	0.390^a^
Preoperative EF (%)		52.5	47.5, 60	50	45, 60	0.999^a^
Postoperative EF (%)		50	45, 55	50	45, 60	0.160^a^
Number of bypasses		3	3, 3	3	3, 4	0.485^a^
Perioperative bleeding (mL)		750	500, 950	725	450, 1000	0.973^a^
Bleeding (24h) (mL)		300	200, 400	300	200, 500	0.896^a^
Total bleeding (mL)		1800	1300, 2300	1775	1200, 2300	0.999^a^
Erythrocyte (unit)		1	0, 2	0	0, 1	0.136^a^
FFP (unit)		2	0, 2	2	0, 2	0.599^a^
Intensive care unit stay (day)		2	1, 4	1	1, 2	0.020^a^
Drain removal time (day)		2	2, 3	2	2, 3	0.994^a^
Clinical admittance (day)		6	6, 8	6	6, 7	0.370^a^
Discharge period (day)		9	7, 14	7	7, 8	0.051^a^
Cross-clamping time (minute)		70	51, 88	48	40, 67	0.040^a^
CPB time (minute)		104	94, 113	91.5	70, 112	0.091^a^

a Mann-Whitney U test;

b Pearson's chi-square test;

d Fisher's exact test;

‡ No measures of association are computed.

CPB=cardiopulmonary bypass; EF=ejection fraction; FFP=fresh-frozen plasma; IQR=interquartile range, reported as first quartile, third quartile; LIMA=left internal mammary artery; RIMA=right internal mammary artery

## DISCUSSION

It is a fact that antiplatelet agents, particularly thienopyridine derivative clopidogrel, are used before PCI in most of the ACS patients^[11,12]^. The majority of the ACS patients to whom surgical revascularization is indicated are candidates for elective CABG surgery while the remaining minority of them need emergency CABG surgery. In a study, 7-12% of patients with no ST-elevation myocardial infarction (STEMI) and 4% of patients with STEMI were reported to undergo CABG surgery during admittance to the same hospital^[13]^. It is also known that some of the ACS patients undergo emergency CABG surgery within 24 hours after diagnosis. Therefore, the protocol recommending discontinuation of clopidogrel 5-7 days before CABG surgery couldn't be applied to these group of patients. In such emergency conditions, considering antiplatelet treatment responsible for postoperative bleedings has been a controversial subject for a long time.

There is not enough data in the literature about whether clopidogrel use must be discontinued or not in case of emergency CABG surgery in ACS patients. Some studies have reported that preoperative clopidogrel use was associated with greater reoperation rates and increase in use of red blood cell and FFP^[14,15]^. Although emergency CABG surgery is required, administration of antiplatelet treatment is an issue that should be evaluated by cardiologists and surgeons. In conditions of bleeding or cardiac events, the interval between treatment discontinuation and CABG surgery is important for the classification of individual risks. This forces cardiologists, anesthesiologists, and cardiovascular surgeons to develop shared decision-making strategies for optimal management of risky patients. Postoperative bleeding makes surgeons anxious while recurrent ischemic events due to discontinuation of antiplatelet agent make cardiologists anxious. Because of the bleeding risk, delaying CABG surgery may increase mortality and morbidity in high-risk patients. In such conditions, complications like excessive perioperative bleeding and excessive use of blood and blood products may be neglected. On the other hand, delaying the operation is another option by discontinuing antiplatelet treatment and taking the risk of recurrent ischemic events. In contrast to guidelines recommendations, it is reported that about 87% of non-STEMI patients receiving clopidogrel in the daily practice and requiring CABG are undergoing surgery within 5 days or less after treatment discontinuation and this increases the need for blood transfusion^[16]^.

In the Clopidogrel in Unstable angina to prevent Recurrent ischemic Events (CURE) trial performed with 1015 patients who had undergone CABG surgery during the same hospitalization period, it was reported that preoperative use of clopidogrel reduced recurrent ischemic events at a rate of 44%, but an increase was observed in the risk of perioperative and postoperative bleedings in patients receiving clopidogrel and ASA within 5 days before surgery^[17]^.

In a meta-analysis study by Biancari et al.^[18]^, the authors reported that the use of clopidogrel before CABG surgery was associated with significant increase in postoperative death, bleeding, reoperation, and need for blood transfusion. Also, in a study by Firanescu et al.^[19]^, the authors reported that there was no difference between discontinuation of clopidogrel 3 or 5 days before surgery regarding postoperative bleeding and use of blood and blood products, but there was a significant difference comparing these patients to those who had undergone surgery without discontinuing clopidogrel regarding postoperative bleeding and blood and blood products replacement.

On the contrary, there are studies stating that there is no difference between patients undergoing surgery while using clopidogrel or not preoperatively. In a study with 322 patients by Kim et al.^[20]^, the authors reported that there was no association between use of clopidogrel within 5 days before CABG surgery and bleeding and reoperation. Also, in a study by Nagashima et al.^[21]^, after comparing groups in which antithrombotic treatment was stopped and not stopped at 5-7 days before CABG, the authors found similar total volumes of intraoperative bleeding and bleeding at postoperative 48 hours between them. Similarly, they stated that they did not find significant difference between drain removal times and lengths of ICU and hospital stay. In the study by Karabulut et al.^[22]^, the authors did not observe significant difference between use of blood and blood products, amount of bleeding, tamponade and reoperation incidences, and lengths of ICU and hospital stay; and they stated that the rates of postoperative homolog blood and FFP use were similar in their patients. Leong et al.^[23]^ observed no significant relationship between receiving clopidogrel and amount of bleeding in patients who had undergone off-pump CABG surgery. In the study by Erdem et al.^[24]^, patients receiving different doses of clopidogrel and undergoing emergency PCI and emergency CABG surgery due to ACS were compared regarding effects of postoperative bleeding, reoperation due to bleeding, and length of ICU and hospital stay on early mortality. When the patients receiving high-dose and low-dose of clopidogrel treatment were compared regarding postoperative chest tube drainage, the authors observed no significant difference. However, they reported a significant difference in patients receiving high-dose of clopidogrel compared to patients not receiving clopidogrel^[24]^.

In our study, when we compared groups receiving or not clopidogrel preoperatively, we could not find a significant difference between the amount of perioperative and postoperative bleeding, rates of reoperation, and tamponade (*P*>0.05; Table 2). We also found similar drain removal times and lengths of ICU and hospital stays (*P*>0.05). In patients receiving clopidogrel preoperatively, while the difference between amounts of erythrocyte and FFP transfusions was not significant, amounts of fresh blood and platelet transfusions were observed to be higher (*P*=0.01, *P*=0.02; Table 2). Namely, we observed that receiving clopidogrel led to an increase in the amount of fresh whole blood and platelet transfusions, but it did not have a significant effect on bleeding and postoperative complications.

Another issue related to clopidogrel is the resistance. It is estimated that 10-15% and 29% of the normal population has resistance to ASA and clopidogrel, respectively. There are also reports indicating that the resistance to aspirin and clopidogrel is approximately 9%^[25]^. Many factors play a role in resistance to clopidogrel, and clopidogrel bioavailability shows variability between individuals^[26]^. Inappropriate dose and drug-drug interactions are other factors affecting the conversion of clopidogrel to its active metabolite. Another important determinant is the genetic polymorphism of the P2Y12 receptor. Certain receptor alleles, ineffective binding to ADP receptor antagonists, and polymorphisms of cytochrome p450 system may also cause resistance. Also, modified risk factors like smoking and stress may play a role^[27]^. Resistance to clopidogrel may cause many disadvantageous conditions in terms of cardiology, such as thromboembolic events. Many studies showed that the determination of platelet reactivity levels played a role in the prediction of bleeding risk and, indirectly, in the surgery timing^[28,29]^. There are studies finding that platelet function tests in the early period are recommendable for guiding treatment discontinuation. It is said that this condition may permit waiting time to be 50% shorter than recommended by guidelines^[30]^.

**Fig. 1:**
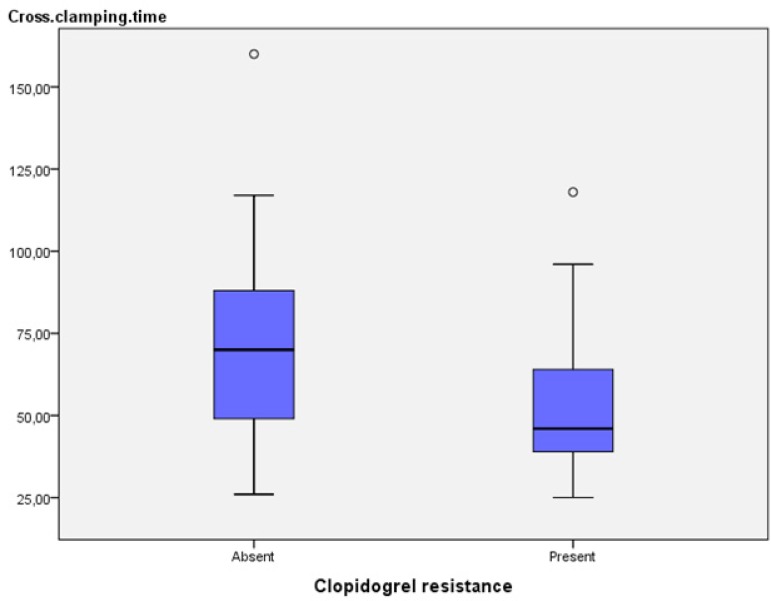
Clopidogrel resistance/cross-clamping time relationship.

In our cases, as indicated earlier, no significant difference was determined between patients receiving clopidogrel and those not, regarding amounts of total bleeding, erythrocyte suspension transfusion, and FFP replacement. We observed that resistance to clopidogrel among patients receiving clopidogrel did not affect the amount of total bleeding, erythrocyte suspension, FFP replacement, and the length of hospitalization (*P*=0.999, *P*=0.136, and *P*=0.599, respectively; Table 3). However, lengths of ICU and cross-clamping times of clopidogrel-resistant patients (Figure 1) (*P*=0.020, *P*=0.040) were observed to be statistically significant (*P*<0.05).

During comparison of patients with and without clopidogrel resistance among the ones receiving clopidogrel, no significant difference was determined between troponin values, preoperative and postoperative EF values, total bleeding volumes during and after surgery, amount of erythrocyte suspension (ERT) and FFP transfusions, and length of hospitalization (Table 3; *P*>0.05).

### Limitations

The limitations of our study are a small number of patients and the fact that it is a single center study. Unfortunately, since there is a great variability in individual response among patients treated with antiplatelet agents, especially with clopidogrel, it is hard to predetermine clearly which patient is exposed to the risk of excessive bleeding and transfusion. It can be difficult to predict only by looking for clopidogrel resistance preoperatively. Keeping in mind that genetic polymorphism of the P2Y12 receptor is also an important factor, it can be useful to perform a meta-analysis of a multicenter genetic study, increasing the samples.

## CONCLUSION

It was observed that the presence or absence of resistance to clopidogrel did not cause a difference in the postoperative period of patients regarding erythrocyte suspension transfusion, FFP replacement, and bleeding-related complications. Also, considering the current costs, we think that resistance studies in patients receiving clopidogrel before cardiac surgery cannot be a routine efficient test to predict bleeding and bleeding-related complications in patients undergoing or who might undergo emergency CABG surgery. We think that it could be useful to hold preparations of fresh blood, erythrocyte suspension, FFP, and platelet suspension on hand as they could be needed in addition to surgical diligence regarding bleeding and bleeding-related complications in patients undergoing emergency CABG surgery while receiving clopidogrel.

**Table:** 

Authors' roles & responsibilities	
MK	Substantial contributions to the conception or design of the work; or the acquisition, analysis, or interpretation of data for the work; final manuscript approval
ZA	Substantial contributions to the conception or design of the work; or the acquisition, analysis, or interpretation of data for the work; final manuscript approval
UV	Substantial contributions to the conception or design of the work; or the acquisition, analysis, or interpretation of data for the work; final manuscript approval
AYB	Substantial contributions to the conception or design of the work; or the acquisition, analysis, or interpretation of data for the work; final manuscript approval
AAA	Substantial contributions to the conception or design of the work; or the acquisition, analysis, or interpretation of data for the work; final manuscript approval
SY	Substantial contributions to the conception or design of the work; or the acquisition, analysis, or interpretation of data for the work; final manuscript approval
